# How much energetic trade‐offs limit selection? Insights from livestock and related laboratory model species

**DOI:** 10.1111/eva.13320

**Published:** 2021-11-28

**Authors:** Frédéric Douhard, Mathieu Douhard, Hélène Gilbert, Philippe Monget, Jean‐Michel Gaillard, Jean‐François Lemaître

**Affiliations:** ^1^ GenPhySE INRAE ENVT Université de Toulouse Castanet‐Tolosan France; ^2^ Laboratoire de Biométrie & Biologie Evolutive CNRS UMR 5558 Université Lyon 1 Villeurbanne France; ^3^ CNRS IFCE INRAE PRC Université de Tours Nouzilly France

**Keywords:** livestock breeding, metabolic rate, pleiotropy, senescence, trade‐offs

## Abstract

Trade‐offs between life history traits are expected to occur due to the limited amount of resources that organisms can obtain and share among biological functions, but are of least concern for selection responses in nutrient‐rich or benign environments. In domestic animals, selection limits have not yet been reached despite strong selection for higher meat, milk or egg yields. Yet, negative genetic correlations between productivity traits and health or fertility traits have often been reported, supporting the view that trade‐offs do occur in the context of nonlimiting resources. The importance of allocation mechanisms in limiting genetic changes can thus be questioned when animals are mostly constrained by their time to acquire and process energy rather than by feed availability. Selection for high productivity traits early in life should promote a fast metabolism with less energy allocated to self‐maintenance (contributing to soma preservation and repair). Consequently, the capacity to breed shortly after an intensive period of production or to remain healthy should be compromised. We assessed those predictions in mammalian and avian livestock and related laboratory model species. First, we surveyed studies that compared energy allocation to maintenance between breeds or lines of contrasting productivity but found little support for the occurrence of an energy allocation trade‐off. Second, selection experiments for lower feed intake per unit of product (i.e. higher feed efficiency) generally resulted in reduced allocation to maintenance, but this did not entail fitness costs in terms of survival or future reproduction. These findings indicate that the consequences of a particular selection in domestic animals are much more difficult to predict than one could anticipate from the energy allocation framework alone. Future developments to predict the contribution of time constraints and trade‐offs to selection limits will be insightful to breed livestock in increasingly challenging environments.

## 1 INTRODUCTION

Most livestock breeding programmes currently generate hyperproductive animals: in less than half a century, the chicken body growth rate has increased almost fivefold (Collins et al., 2014; Havenstein et al., 2003; Zuidhof et al., 2014), annual egg production of layer hens has doubled (Preisinger & Flock, 2000), pig litter size at birth has increased since the nineties by one piglet every 5 years (Merks et al., 2012), and annual milk yield of dairy cows has increased by 140 kg every year from the seventies. As selection for higher productivity (see glossary in Box 1 for a definition) generally accelerates the conversion of feed supply into increasing amounts of animal products (Vandehaar, 1998), it has been a successful economic strategy to make nutrient‐rich animal source food increasingly affordable to a growing human population (Garnett et al., 2013). Besides the major sustainability concerns including environmental, health and welfare issues, the large and continuous increase in livestock productivity falls into the lively debate regarding biological limits to energy expenditure (Denny, 2008; Piersma, 2011; Careau et al., 2013; see Marck et al., 2017 for a review).

BOX 1 **Glossary**

*Basal metabolic rate (BMR):* the lowest measurable rate of energy expenditure in a nonreproducing adult endotherm, during inactive, postabsorptive and not‐growing stages (McNab, 1997).

*Breeding objective (or goal):* traits to be improved in a breeding programme and the relative emphasis that is given to each trait. This sets the direction of artificial selection.

*Feed efficiency:* productivity output (expressed in mass, energy or protein) relative to the amount of feed consumed (i.e. input). Feed efficiency metrics are generally defined either as a ratio (i.e. output/input, the feed conversion ratio) or as a measure of feed intake adjusted for production requirements (e.g. residual feed intake; see below). *Feed* for livestock is distinguished from *food*, that is feed for human consumption. Edible crops can be used as feed or food so improving feed efficiency may contribute to reduce feed–food competition.

*Genetic gain:* for a particular trait, it is the change in average breeding value between one generation of selection candidates and the next generation only formed from the candidates that were selected and became parents.

*Metabolizable energy intake (MEI):* the amount of energy that an animal assimilates from feed intake on a daily basis. MEI is usually calculated as the amount of feed intake times the metabolizable energy density of the diet.

*Productivity:* the production of new biomass via growth or reproduction and measured as growth rates or rate of production by adult females (e.g. offspring, eggs, milk; Biro & Stamps, 2010). Energy allocation to productivity includes the energy contained in new biomass and the energy used for this biosynthesis and dissipated as heat. Productivity traits reflect production performed during a given time period that is determined either biologically (e.g. pig litter size at birth is accumulated during gestation) or technically (e.g. cow annual milk yield usually refers to a standard lactation period of 305 days). A major outcome of animal breeding has been the generation of breeds or lines hyperspecialized in particular types of productivity (e.g. meat‐type fast‐growing vs. egg‐type in poultry, beef vs. dairy in cattle).

*Physiological constraints:* the lack of genetic variation in physiological control mechanisms of a productivity trait that prevents further selection (Ricklefs & Wilkelski, 2002).

*Residual feed intake (RFI):* a measure of feed intake adjusted for production requirements that corresponds to the difference between actual feed intake and feed intake predicted from body mass and actual production (e.g. mass gain, milk yield over the period on which feed intake is measured). Individuals with lowest RFI are consistently deemed to be the most efficient.

*Resting metabolic rate (RMR):* the lowest metabolic rate of an endotherm when one or more of the conditions required for measuring BMR cannot be met (i.e. during adult, postabsorptive, nonreproductive or resting phase; Careau & Garland, 2012). When animals are in a production phase, RMR includes the energy costs to sustain the production processes (e.g. enlargement of central organs involved in energy supply such as the intestines, liver, kidney or heart, and increase in their metabolic activity; Biro & Stamps, 2010).

*Self‐maintenance:* the different vital processes involved to maintain an animal in a state of energy equilibrium implying nor loss or gain when no energy is required for activity, growth or reproduction (Knap, 2009). In practice, maintenance energy requirements for self‐maintenance can be reasonably approximated by RMR under laboratory experimental conditions. Energy allocation to self‐maintenance contributes to soma preservation and repair. It thus favours future survival and reproduction and delays senescence (Kirkwood, 2017).

**FIGURE 1:**
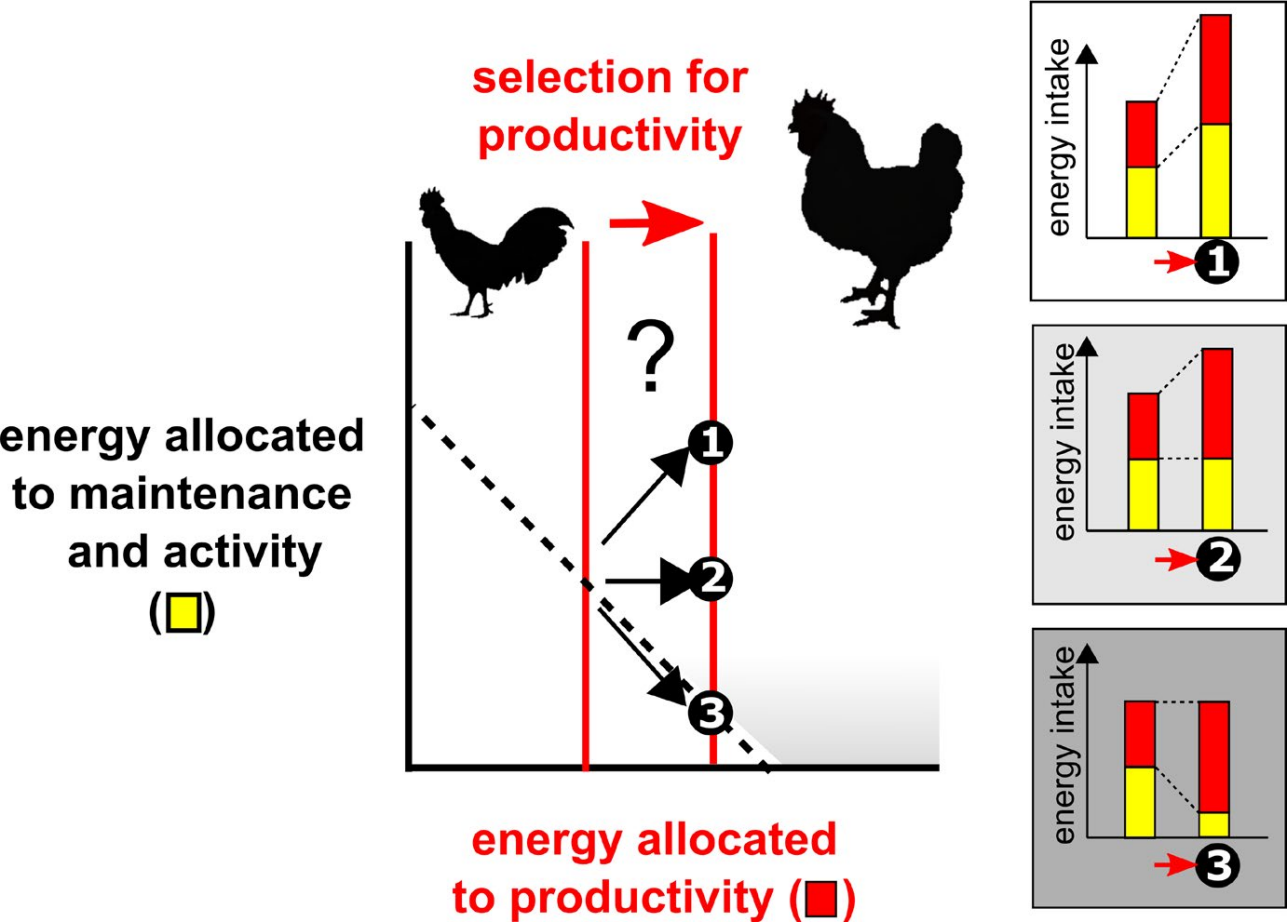
Several paths of change in energy acquisition and allocation may be expected from applying the Y model of energy allocation to selection for livestock productivity. MEI allocated to productivity (energy deposited in new biomass during growth or reproduction plus the energy of biosynthesis; in red) versus maintenance and activity (once the covariation with body size has been accounted for; in yellow). Each point represents an individual, and paths 1 to 3 predict how a same selective increase in a productivity trait (e.g. growth rate) would be mediated through energy acquisition and allocation depending on the energy available. Grey shade reflects the trade‐off intensity

*Trade‐off:* when one trait cannot increase without a decrease in another trait (or vice versa; e.g. Garland, 2014). If an increase in the expression of a trait requires more energy and if this need of extra‐energy implies a reduction in another component of the energy budget, then an energetic trade‐off occurs (Careau & Garland, 2012), as in the Y model of energy allocation (see path 3 in Figure 1). Energetic trade‐offs are expected to occur when the amount of energy available to organisms is limiting or when organisms are constrained by their time to acquire and process energy.

In agricultural sciences, the extent to which further genetic gains (Box 1) can be sustained is a long‐standing issue (Collier et al., 2005; Fredeen, 1984; Hammond, 1947; Hunton, 1984; Kennedy, 1984; Legates, 1967; reviewed in Hill & Bünger, 2004) that continues to be puzzling (Hill, 2016; Tallentire et al., 2018). Evolutionary theory predicts the depletion of additive genetic variance as a consequence of selection (Hill, 2016; Merilä et al., 2001). Thus, a strong and sustained directional selection on productivity traits should lead to the fixation of favourable alleles and elimination of disfavoured alleles, and thereby should stop the evolution of more productive genotypes (Robertson, 1960). However, most long‐term (i.e. >30 generations) selection experiments in vertebrates have dismissed the existence of limits for various productivity traits, including body growth (Marks (1996) in Japanese quails, Siegel (2014) in meat‐type chicken, Nestor et al. (2008) in turkey and Bünger et al. (2001) in mice), egg production (Poggenpoel et al. (1996) in chicken) or litter size (Estany et al. (1989) in rabbits, Holt et al. (2005) in mice, and Hsu and Johnson (2014) in pigs). These results match observations from populations of animals in the wild and humans for which genetic variation also remains high for many traits that are under directional or stabilizing selection (Merilä et al., 2001; Polderman et al., 2015). Moreover, the existence of selection limits for expensive behaviours in mice (e.g. nest‐building, voluntary wheel‐running) indicates that the depletion of additive genetic variance in the selected trait is not necessarily involved (Bult & Lynch, 2000; Careau et al., 2013).

Selection for productivity can result in correlated responses in other traits, which in return may have detrimental effects on fitness (Lerner & Dempster, 1951). For at least three decades, the continuous increase in productivity within livestock populations has been accompanied by negative side effects on health and fertility traits (Berry et al., 2014; Julian, 1998; Nebel & McGilliard, 1993; Rauw et al., 1998; Roberts, 1979). To counterbalance these negative side effects, several affected traits are now part of the breeding objectives (Box 1) as their continuous degradation would otherwise carry economic losses (Hayes et al., 2013; Miglior et al., 2005; Neeteson‐van Nieuwenhoven et al., 2013). However, it remains unclear whether selection adjustments in the breeding objectives are high enough to mitigate the degradation in health, survivorship or fertility (Berry et al., 2014) or to prevent the occurrence of new detrimental effects (e.g. increasing incidence of growth‐related breast meat abnormalities in chicken selected for fast growth; Soglia et al., 2018). Neither do we know whether those negative side effects are linked to physiological constraints (Box 1), which may ultimately impose limits to selection (Blows & Hoffmann, 2005).

In all organisms, productivity measures the output of an energy conversion process governed by general physical and chemical principles and sustained by a metabolic engine that is also energetically costly to maintain (Biro & Stamps, 2010). Accordingly, the traditional view that the energy available to individuals is limited so that any energy allocation to one function reduces the allocation to another function (Cody, 1966) has also been considered in the context of livestock and laboratory animals (Beilharz et al., 1993; Rauw, 2009). The resource allocation theory became popular within livestock sciences after Rauw et al. (1998) conjectured that it offers a biological explanation for the widespread occurrence of negative side effects of selection for high productivity. This view received some support from experimental comparisons of selected lines (e.g. Rauw et al., 1999; Savietto et al., 2014; Schütz & Jensen, 2001; Theilgaard et al., 2007) and was further established through reports on farm animal health and welfare (e.g. Oltenacu & Broom, 2010; Prunier et al., 2010). Although developments of a resource allocation framework in the livestock context have then focused on predicting the consequences of animal breeding (Doeschl‐Wilson et al., 2009; Friggens & Newbold, 2007; Van Der Waaij, 2004), the applicability of the allocation principle to such context has rarely been questioned (Gilbert et al., 2017). This may seem confusing as environments optimized for production such as farms or laboratories are unlikely to primarily set limits on the amount of energy that animals can obtain. Without such limits and given the persistent genetic variation in productivity traits, livestock selection should simply generate animals that escape resource allocation trade‐offs by compensating the higher nutrient allocation to biosynthesis with higher rates of feed acquisition (van Noordwijk & de Jong, 1986; Reznick et al., 2000). Yet, in practice, nutrient intake may be frequently limited by the economic dependence of intensive production systems on expensive nutrient‐rich feed inputs such as grain (Garnett et al., 2013). Nutritional limitations may also be imposed by farmers to avoid health problems of excess nutrient consumption (D’Eath et al., 2009) or may occur incidentally (e.g. as a result of competitive group‐feeding; Grant & Albright, 2001).

Most obviously, time must be limiting and time constraints remain independently of the amount and quality of resources available (Lemaître et al., 2020), leading to the existence of potential limits in the rate at which animal bodies metabolize and process energy (Hammond & Diamond, 1997). Animals selected for high productivity may increase their time spent foraging but such increase is inevitably limited. For instance, dairy cows selected for high milk yield spend an increasing proportion of the day eating at the expense of lying time which, in turn, may approach welfare limits (Løvendahl & Munksgaard, 2016). There are also inherent physiological limits on the time to achieve certain production processes. For instance, layer hens cannot produce more than one egg per day under a normal light–dark cycle of 24 h, so selection on egg production basically seeks to increase the laying frequency over the annual production cycle (Hunton, 1984). Such time constraints are exceptionally challenged in livestock breeding given that feed availability is relatively nonlimiting but that strong selection is exerted to quickly convert feed intake into animal products. However, in those conditions where animals are mostly constrained by their time to metabolize energy rather than by the amount of energy, we still have a limited knowledge of how do selective increments in productivity translate in terms of energy allocation trade‐offs (Jackson & Diamond, 1996; Konarzewski et al., 2000). Although the role of time constraints and subsequent energy allocation in life history evolution is well established (Arnold, 1992; Garland & Carter, 1994; Ricklefs & Wilkelski, 2002), their link to the limits of selection for higher rates of energy expenditure in the form of animal products is mostly unexplored, despite their strong biological and socio‐economic implications.

Here, we propose that the apparent absence of selection limits for increased productivity despite the existence of physiological trade‐offs illustrates a conundrum in our understanding of selection responses. Did selection for ever‐increasing rates of biosynthesis in nutrient‐rich environments lead us to overlook the existence of physiological limits to acquire and process energy, which can then favour the occurrence of energy allocation trade‐offs? To address this issue, data from livestock and related laboratory models represent a relevant starting point. Indeed, a large body of research on feed efficiency (Box 1) of agricultural production exists that is primarily motivated by economic implications as feed accounts for most of the production costs on farms (Johnson et al., 2003). Greater feed conversion rates or feed efficiency is limited by the energy required for animal maintenance, which goes from 10% in pigs and poultry to more than 50% in ruminants, whereas this energy allocated to self‐maintenance (Box 1) is thought to be key to sustain production processes over lifetime (Kirkwood, 2017). This research on livestock feed efficiency has a clear general biological basis, which, for instance, allowed inference from laboratory species such as mice or Japanese quails (Pitchford, 2004). However, its evolutionary implications have been seldom considered (Swallow et al., 2009). Though, data available so far represent an untapped opportunity to highlight the role of energetic trade‐offs in selection responses across diverse vertebrate species (Swallow et al., 2009). Here, we predict that if the energy allocation framework strictly applies, then selection for high productivity early in life should concurrently reduce the relative energy allocation to self‐maintenance (*prediction 1*). Those changes in the energy allocation to productivity relative to the allocation to maintenance are more direct when selecting for productivity outputs while controlling for feed inputs. In this last case, we thus expect a reduced energy allocation to maintenance and repairing processes that will result in impaired health or future reproduction (*prediction 2*). After examining those predictions with the data currently available in the literature, we argue that the mechanisms through which productivity is selectively increased should be addressed in a more complete energy allocation framework than the current one. This appears as a key issue if we are to predict the consequences of selection for livestock bred in increasingly diverse and challenging environments.

## 2 TRADE‐OFFS DEFINED IN THE ENERGY ALLOCATION FRAMEWORK

The principle of energy allocation between self‐maintenance, growth and reproduction is a cornerstone of life history theory on which the expectation of evolutionary trade‐offs is based (Cody, 1966; Williams, 1966). Accordingly, several studies performed in the wild have revealed negative associations between the allocation to growth or reproduction and the subsequent survival or reproductive performance (Hamel et al., 2010; Lemaître et al., 2015). However, trade‐offs are far from being consistently detected and positive associations are commonly reported (Lemaître et al., 2015, 2020). Van Noordwijk and de Jong (1986) proposed that energy allocation trade‐offs should only occur when individual variation in resource allocation is larger than the variation in resource acquisition (a model called the ‘Y model’). However, the model assumption that the relative resource allocation is independent from resource acquisition is too restrictive to cover the diversity of resource allocation strategies observed among taxa (Descamps et al., 2016). Moreover, the association between resource acquisition and resource availability is complex and prevents any reliable prediction of whether a trade‐off occurs or not in a particular context (Boggs, 2009; Glazier, 1999; Roff & Fairbairn, 2007). In practice, energy acquisition and allocation are difficult to measure and are most commonly inferred from a limited number of phenotypic measurements (Ng’oma et al., 2017). Experimental manipulations of the quantity or quality of feed supply or of the reproductive effort have shown that physiological trade‐offs are more acute under stressful environments (Boggs, 2009). Likewise, several studies in the wild have detected fitness cost of reproduction only when resources are limited during specific years and/or in specific areas (Cohen et al., 2019). For example, in Soay sheep (*Ovis aries*), young breeding males suffer from an impaired survival only in years of high population density (Stevenson & Bancroft, 1995), likely as a result from an earlier allocation to reproduction. The costs of reproduction are indeed particularly high in this species, probably because Soay sheep bear the hallmarks of selection for increased productivity during their initial domestication (Clutton‐Brock & Pemberton, 2004). Still, in the wild, a proper test of the Y model is largely hindered by practical difficulties to get reliable estimates of individual variation in resource acquisition. This is less of an issue in high‐input livestock production systems where the availability of feed intake data enables large‐scale genetic evaluations.

In contrast to life history evolution that primarily views the principle of allocation as a powerful theoretical framework to interpret evolutionary outcomes, this principle has been more narrowly applied to livestock breeding as an organismal constraint on the fluxes of energy and nutrients between functions that governs responses to artificial selection (Beilharz et al., 1993; Rauw, 2009). Under the Y model, MEI (Box 1) can be split on a daily basis by individuals into one part allocated to productivity (energy retained as new biomass such as muscle, fat, milk, egg or wool plus the associated energy of biosynthesis dissipated as heat; Figure 1), and the other part allocated to activity and self‐maintenance (with the latter maximizing the chance of survival and future reproduction; Kirkwood, 2017; Box 1). Most negative side effects of selection for high productivity are thus thought to stem in the extent to which energy allocation to maintenance is reduced in the total energy budget MEI (Beilharz et al., 1993; Rauw et al., 1998) once its covariation with body size has been accounted for (Konarzewski & Książek, 2013). However, under the assumption of independence between resource acquisition and relative allocation (Descamps et al., 2016), a same increase in productivity over generations of selected animals could be mediated through an increase in MEI, or through an increase in relative allocation (i.e. energy allocation to productivity relative to MEI), or through a mixture of both changes possibly independently from each other (Figure 1).

Further, if we consider that energy allocation to maintenance mostly reflects the minimal energetic cost to maintain the tissues and essential life functions or to support the ‘metabolic engine’ (Biro & Stamps, 2010), then the different paths of Figure 1 match hypotheses developed by physiologists to explain intra‐specific variation in BMR (Box 1) in an ecological context (Burton et al., 2011; Careau & Garland, 2012). Similar to the ‘independent hypothesis’ that predicts variation in metabolic rates independently from daily energy expenditure (Konarzewski, 1995), livestock nutrition generally considers that energy allocation to productivity is independent from the allocation to maintenance (path 2 in Figure 1). A key implication of this assumption is a dilution of the maintenance energy requirements (Bauman et al., 1985; Vandehaar, 1998) so that gains in livestock productivity are associated with gains in feed efficiency (Capper & Bauman, 2013). In other words, the energy costs of maintenance are assumed to be fixed, leading the maintenance cost per unit of animal production to be reduced. In contrast, a greater productivity might involve higher maintenance costs (the ‘increased‐intake hypothesis’, Biro & Stamps, 2010; Burton et al., 2011; Careau & Garland, 2012; Koteja, 2000; Nilsson, 2002; path 1 in Figure 1).

Studies in livestock have implicitly assumed that the amount of feed available in the environment (often categorized as ‘limiting’ vs. ‘nonlimiting’) determines which of the previous paths is undertaken during selection for increased productivity. In limiting environments (path 3 in Figure 1), trade‐offs show up because any increase in productivity should result in reduced energy allocation to maintenance during the productive phases of animal life (cf. prediction 1). If this reduction is associated with insufficient maintenance and repairing processes, then the capacity to start over a new productive cycle or to remain healthy should be impaired (cf. prediction 2). In contrast, energy allocation trade‐offs are unlikely to occur as far as the feed supply keeps up with genetic gains in productivity (paths 1 and 2 in Figure 1), unless the levels of productivity approach physiological limits stemming from time constraints. Indeed, under those conditions we contend that time constraints on energy allocation should be prominent and thus lead energy allocation trade‐offs to occur as predicted under limited energy availability (cf. predictions 1 and 2). Most evidence for the previous framework accumulated so far is indirect or correlative, and its predictions have not been formally tested in livestock or laboratory animals.

## 3 SURVEY OF BREED COMPARISONS AND SELECTION EXPERIMENTS

To assess prediction 1 (i.e. selection for productivity results in reduced allocation to maintenance) and prediction 2 (i.e. reduced energy allocation to maintenance results in impaired health or future reproduction), we surveyed studies in livestock species (mammals and birds) that have investigated the genetic relationships between productivity and energy allocation to self‐maintenance. We thus performed a systemic search in Web of Science by using the following string of search terms: (‘metabolic rate’ OR ‘maintenance’ OR ‘heat production’) AND (‘line’ OR ‘breed’ OR ‘strain’) in the Web of Science categories (‘Agriculture Dairy Animal Science’ OR ‘Genetics Heredity’ OR ‘Veterinary Sciences’ OR ‘Ecology’ OR ‘Multidisciplinary Sciences’), and using citation chaining (Figure S1 and the PRISMA diagram in Supplementary Material). Surprisingly, eligible studies were quite rare. We looked chiefly for selection experiments as most of the phenotypic differences among lines can be attributed to genetic differences generated during the selection process. Moreover, selection experiments are particularly relevant to investigate physiological constraints in action during evolutionary changes (Garland, 2003; Box 2). Unfortunately, only a few of them have actually measured RMR in livestock. Data on RMR are more frequent for a particular breed or commercial line to estimate its nutritional requirements. In this case, the genetic effect related to productivity was less obvious, but our survey also included those studies based on breed comparisons to further discuss the effect of different levels of productivity on RMR (Tables 1 and 2). To gain complementary insights, we also considered laboratory species that have been commonly used in livestock research as relevant biological models. Thus, results from previous syntheses within particular taxa (e.g. Konarzewski & Książek, 2013) could be compared with those available for other mammals and birds.

**TABLE 1:** Comparison of energy allocation to maintenance among lines or breeds differentially selected on a growth trait

Species	Protocol^a^	Selection line criteria or breed	Compared with	Method RMR^b^	Allometry^c^	Age or mass	RMR	Fatness	Reference^d^
Mice	SE	High body mass gain (3–6 w)	Unselected line	CS	/m^0.75^	3–6 w	n.s.	+	G1
	SE	High fatness (10 w)	Unselected and opposite lines	RE	/m^0.75^	3–10 w	n.s.	[+]	G2a
		High lean body mass (10 w)	Unselected and opposite lines				n.s.	n.s.	G2b
	SE	High body mass (6 w)	Unselected line	IC	/m^0.75^	4, 7 w	−	+	G3a
		High carcass protein (6 w)	Unselected line				n.s	n.s	G3b
Japanese quail	SE	High body mass (6 w)	Unselected and opposite lines	IC	/m^0.75^	3 w	−	+	G4
Chicken	SE	High body mass (8 w)	Opposite line	IC	/m	4, 6, 8 w	−	NA	G5
	SE	High body mass gain (5 to 9 w)	Unselected line	IC	/m	5 w	−	n.s. (9 w)	G6
	LC	Fast‐growing meat‐type strain	Taiwanese native strain	RE	/m^0.75^	1–4 w	n.s.	+	G7
	LC	Fast‐growing meat‐type strain	Slow‐growing egg‐type strain	IC	cov (m lean)	40–80 g 80–160 g	− n.s.	+ −	G8
	LC	Fast‐growing meat‐type strain	Slow‐growing egg‐type strain	IC	cov (m)	1–8 w	−	NA	G9
	LC	Fast‐growing meat‐type strain	Red junglefowl	IC	cov (m)	0–9 w	n.s.	NA	G10
Turkey	SE	High body mass (16 w)	Unselected line	IC	/m	1 w	n.s.	NA	G11
Pig	SE	High body mass gain and low fatness	Low body mass gain and high fatness	IC	/m^0.75^	40–90 kg	+	[−]	G12
	LC	Large‐sized breed (Landrace)	Medium‐sized lean breed (Duroc)	RE	/m^0.75^	58 kg	+	n.s.	G13
	LC	Fast‐growing large‐sized breed	Medium‐sized lean breed	IC	/m^0.75^	10 w 17 w 24 w	n.s. n.s. +	n.s. n.s. n.s.	G14
	LC	Fast‐growing lean breed (Large White), castrate	Slow‐growing fat breed (Meishan), castrate	IC	/m^0.60^	25, 40, 60 kg	+	[−]	G15
Goat	LC	Fast‐growing meat‐type crossbred Boer castrate	Landrace meat‐type Spanish castrate	CS	/m^0.75^	22 w	n.s.	NA	G16
Cattle	LC	High postweaning growth line	Unselected animals from a commercial herd	CS	/m^0.75^	14–18 mo	n.s.	+	G17
	LC	High body mass postweaning	Unselected line	CS	/m^0.75^	15 mo	+	−	G18
	SE	High growth rate	Unselected and opposite lines	RE	/m^0.75^	5–6 yr	−	+	G19

Note

Associations between growth and maintenance are classified as positive (+), negative (−) or not statistically significant (n.s.). Associations in square brackets ‘[]’ indicate direct responses to selection. RMR = resting metabolic rate; age is expressed in weeks (w), months (mo) or years (y).

^a^ SE = selection experiment, LC = comparison of independent lines or breeds.

^b^ CS = comparative slaughter technique, IC = indirect calorimetry, RE = estimation of retained energy from observed changes in body mass and energy intake.

^c^ RMR divided by body mass (/m) or scaled‐body mass (e.g. /m^0.75^), or adjusted through covariance analysis (e.g. cov(m) where body mass (m) is a covariate of RMR).

^d^ G1: Canolty and Koong (1976); G2: Bishop and Hill (1985); G3: Klein et al. (1999); G4: Maeda et al. (1994); G5: Owens et al. (1971); G6: Pym et al. (1984); G7: Lin et al. (2010); G8: Konarzewski et al. (2000); G9: Kuenzel and Kuenzel (1977); G10: Jackson and Diamond (1996); G11: Fan et al. (1997); G12: Sundstøl et al. (1979); G13: Kolstad and Vangen (1996) (re‐evaluated in Knap (2009)); G14: Tess et al. (1984); G15: van Milgen et al. (1998); G16: Tovar‐Luna et al. (2007); G17: Castro Bulle et al. (2007); G18: Batalha et al. (2021); and G19: Herd (1995).

**TABLE 2:** Comparison of energy allocation to maintenance among lines or breeds differentially selected on a maternal reproductive output

Species	Protocol^a^	Selection line criteria or breed	Compared with	Method RMR^b^	Allometry^c^	Age or mass	RMR	Fatness	Reference^d^
Japanese quail	SE	High egg size corrected for female body size	Low egg size corrected for female body size	IC	cov (m)	Adult breeding	n.s.	n.s.	R1
						Adult nonbreeding	n.s.		
Chicken	LC	High egg production lines (White Leghorn breed)	Moderate egg production breed (Rhode Island Red)	IC	/m^0.75^	16 to 64 weeks of age during laying	+	−	R2
Mice	SE	Litter size at birth	Control line	CS	/m^0.73^	Adult (≥60 days)	+	NA	R3
Cattle	LC	Dairy‐type angus crossbred)	Beef‐type angus crossbred	RE	/m^0.75^	Adult (9 year) nonreproducing	+	n.s.	R4
	LC	Dairy breeds	Beef breeds	CS	/m^0.75^	Adult (>10 year) nonreproducing	+	n.s./+	R5
	LC	Angus crossbred (high or medium dairy)	Angus crossbred (low dairy)	RE	/m^0.75^	Adult (6–8 year) during gestation lactation	+	–	R6

Note

Associations between reproductive output and maintenance are classified as positive (+), negative (−) or not statistically significant (n.s.). RMR = resting metabolic rate; age is expressed in weeks (w), months (mo) or years (yr).

^a^ SE = selection experiment, LC = comparison of independent lines or breeds.

^b^ CS = comparative slaughter technique, IC = indirect calorimetry, RE = estimation of retained energy from observed changes in body mass and energy intake.

^c^ RMR divided by body mass (/m) or scaled‐body mass (e.g. /m^0.75^), or adjusted through covariance analysis (e.g. cov(m) where body mass (m) is a covariate of RMR).

^d^ R1: Pick et al. (2016); R2: Bentsen (1983); R3: Rauw et al. (2000); R4: Ferrell and Jenkins (1984); R5: Solis et al. (1988); and R6: Montano‐Bermudez et al. (1990).

BOX 2 **When selection experiments reveal the importance of physiological constraints in selection responses**

**FIGURE 2:**
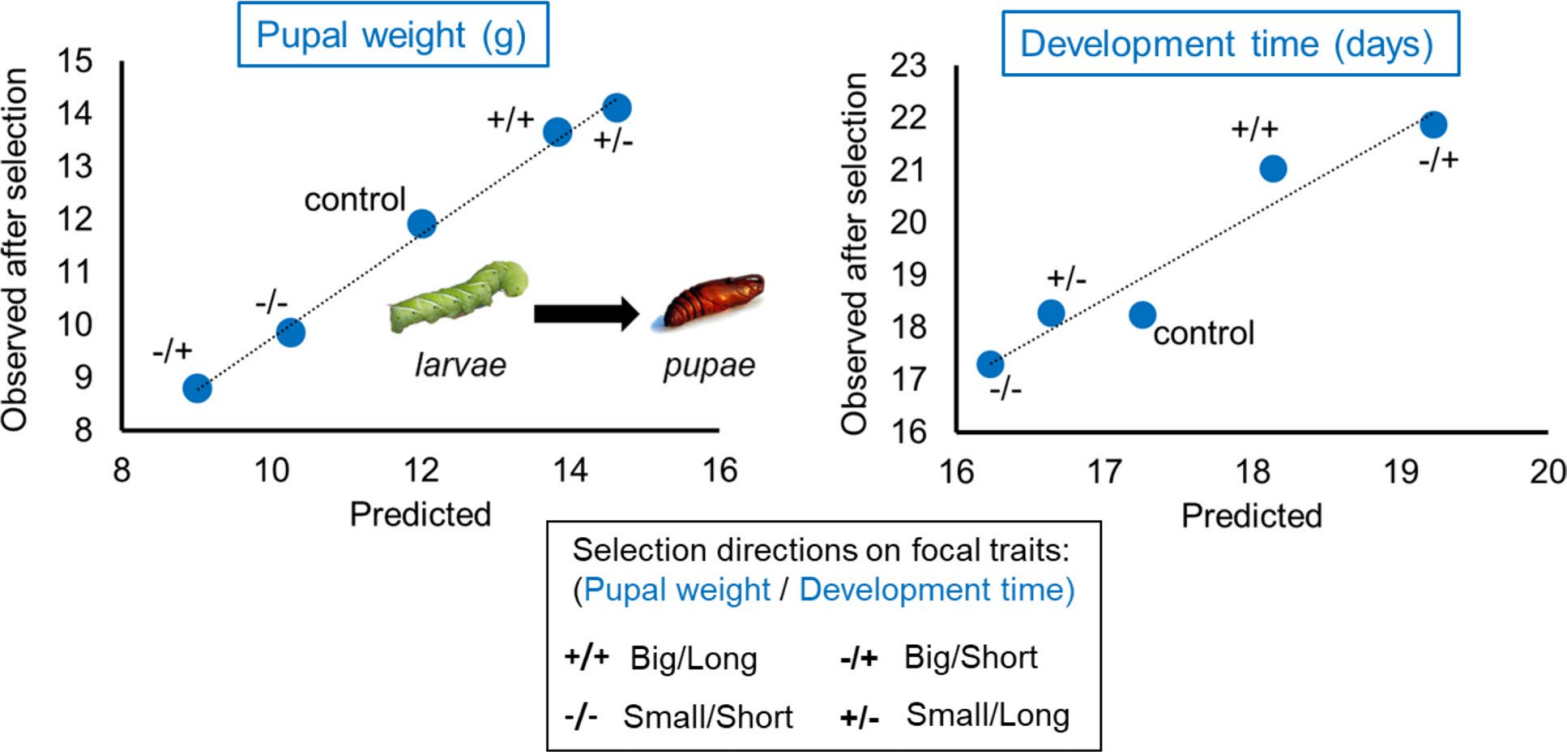
In four different directions of selection on two focal traits of larva development to pupae (pupal weight and development time) in *Manduca sexta* (Sphingidae), direct responses are predictable from fixed relationships between three mechanistic traits describing individual development. Data from Davidowitz et al. (2016). Image sources: Larva image modified from an image by Daniel Schwen available from Wikimedia Commons under licence CC BY‐SA 4.0. Pupa image modified from an image by 7 and available from Wikimedia Commons under licence CC BY‐SA 3.0

Identifying physiological constraints at the level of genes is challenging. Focal traits under selection, such as productivity traits, are often the end products of complex genetic, physiological, developmental and endocrine processes. The biological complexity in the mapping of genotype to phenotype can be reduced by focusing on mechanistic traits, which usually refer to a small number of physiological traits that determine focal traits. To assess the extent to which those mechanistic traits constrain or enable selection responses, laboratory selection experiments exploring multiple directions of selection are relevant tools (Garland, 2003). If in all directions of selection, observed direct responses of focal traits are consistent with predicted responses based on mechanistic traits, this would support strong physiological constraints. This is well illustrated by Davidowitz et al. (2016)’s study case on the tobacco hornworm (*Manduca sexta*; Figure 2).

Two focal traits of larva development to pupation were artificially selected: development time and pupal weight. Those traits can be predicted from their functional relationships with three mechanistic traits (i.e. critical weight (CW), length of the growth period (ICG) and growth rate (GR)) that characterize the last stage of larva development into pupae:

Predicted pupal weight = CW + (ICG × GR),

Predicted development time = ICG + (CW/GR),

where the attainment of the CW marks the transition from the juvenile growth to the reproductive adult phase (initial adult weight), ICG corresponds to the duration of the period of growth from CW to pupal weight, and GR refers to the linear rate of change in body weight during this stage.

Both CW and ICG have positive effects on development time and pupal weight, whereas GR has a positive effect on pupal weight and a negative effect on development time. Pupal weight and development time were simultaneously selected to explore four directions (i.e. same or opposite directions). Regression lines in Figure 2 show that whatever the direction of selection, responses are highly predictable from the correlated changes in the three mechanistic traits. In other words, those physiological determinants of development at the individual level strongly constrain responses to selection in multiple directions.

### 3.1 Prediction 1: selection for productivity results in reduced allocation to maintenance

We distinguished two main types of productivity traits: those related to body growth (Table 1; studies ‘G*i*’), and those related to maternal reproductive outputs such as litter size or milk yield in mammals (Table 2; studies ‘R*i*’). As for energy allocation to self‐maintenance, it cannot be approximated by BMR when animals are in a production phase. We considered instead any measure of RMR (Box 1) as previously done (Biro & Stamps, 2010; Burton et al., 2011). In livestock studies, related metrics include heat production after fasting (using indirect calorimetry) or estimates of energy intake by extrapolation at various feeding levels towards zero energy retention (using comparative slaughter technique to estimate the retained energy (RE) in body mass gain). None of the methods that were developed to determine energy maintenance requirements during productive phases have gained broad acceptance due to the difficulty to tease apart the energetic costs related to productivity from the minimal energetic cost of living (Knap, 2009). Moreover, previous metrics are not homogeneously adjusted for the effect of metabolic allometry that stands within species (Konarzewski & Książek, 2013). Most studies in livestock research divide them by the so‐called metabolic body mass (i.e. m^0.75^), but we also found studies that simply reported mass‐specific estimates and others that used covariance analysis with body mass as covariate, as recommended by integrative physiologists (Konarzewski & Książek, 2013). All studies from our survey used nutrient‐rich diets that were provided *ad libitum*, and thus, MEI was not limited by the environment.

Overall, about half cases (10 out of 21) did not detect any difference in maintenance energy metabolism between animal groups with contrasting growth rates (Table 1). However, results varied across species. In mice, no association was reported between productivity and RMR in 80% of the cases. In poultry, a negative association occurred in two thirds of the cases. In pigs, a positive association was detected in all the four studies, regardless of a selection on low fatness in addition to high productivity (studies G12 and G15). Finally, there was no clear trend in ruminants. Interestingly, the relationship between maintenance and fatness (studies G12, G15 and G18) was reciprocally observed in some cases (i.e. a higher fatness associated with a lower maintenance; studies G3, G4, G8 and G19), and none of the 21 cases indicated a positive covariation between fatness and RMR.

All six studies in reproducing females (Table 2) found a positive effect of maternal productivity on RMR, in support of the ‘increased‐intake hypothesis’ (Figure 1; path 1).

Associations between the genetic potential for growth and maintenance requirements were all measured in growing animals, sometimes in several growth phases (e.g. studies G8 and G14). However, there was little information on the persistence of those associations beyond the growth period in these determinate growers (i.e. few studies on mature animals such as G19). In contrast, evidence for positive associations between maternal productivity and RMR was also observed when adult females were not reproducing (e.g. studies R3–R5). Still, the detection of associations is inevitably hindered by the limited data relative to the large number of factors, notably caused by the use of various metrics to measure RMR.

### 3.2 Prediction 2: reduced energy allocation to maintenance results in impaired health or future reproduction

To study the consequences of genetic changes in the energy allocation to maintenance on components, or physiological correlates, of health or future reproduction, we surveyed selection experiments directly targeting energy metabolism (e.g. Konarzewski et al., 2005) or indirectly through feed efficiency (Table 3, studies ‘M*i*’). Indeed, in the livestock context, feed efficiency and related metrics such as residual feed intake (Box 1) are more commonly studied than RMR because they have higher practical relevance and become increasingly available in commercial populations with the development of individual feed intake monitoring. In our survey, all the selection experiments on feed efficiency where a RMR‐related metric was measured showed that feed efficiency and RMR were consistently and positively related (Table 3). Selection for feed efficiency also affects components other than energy metabolism (e.g. digestive efficiency, especially in ruminants). However, experiments in mice show that, in turn, selection for RMR (e.g. heat loss/m^0.75^ (Exp. M7) or mass‐independent BMR (Exp. M8)) has a positive selection response on feed intake (Table 3). Thus, we assumed that results from selection for high feed efficiency and for low RMR were comparable. Overall, feed efficiency metrics have a moderate heritability (e.g. mean of 0.25 from 35 estimates in seven species as reported by Pitchford, 2004), close to that reported for RMR (Konarzewski & Książek, 2013; Pettersen et al., 2018). Both feed efficiency and RMR metrics thus well respond to selection.

**TABLE 3:** Summary of main responses observed in selection experiments on metabolic rate or feed efficiency in livestock and laboratory models

Species	Selection criteria^a^	RMR	Body mass	Feed intake	Activity	Effects on components or physiological correlates of health (in red) or reproduction (in blue)		Reference^b^
Japanese quail	FI/mass gain (1 to 4 w)		−	[+]				M1
Chicken (egg‐type)	FI cov(m^0.5^, mass gain, egg mass) (33–37 w)	n.s./+	n.s.	[+]	+	+	Humoral immune response cellular immunity traits (heterophils, macrophages)	M2
						n.s.	Age at first egg, egg mass, egg number	
						−	Male fertility, sperm concentration, motility, embryo survival cellular immunity traits (lymphocytes)	
Chicken (meat‐type)	O_2_ consumption/m (3 w)	[+]	− (8w)			+	Egg number, hatching success	M3
						n.s.	Fertility, egg mass	
	FI (5 to 9 w)	+	+	[+]				M4
	FI/mass gain (5 to 9 w)	+	−	n.s.				
Mice	FI cov(m) (4 to 6 w)	+	+	[+]		+	Ovulation rate, litter size number of pups over lifetime	M5
						n.s.	Prenatal survival female adult survival	
	FI cov(m) (8–10 w)	+	n.s. + (18–20 w)	[+]	+	+	Milk energy output, litter growth rate	M6
						n.s.	Oxidative stress (damage and antioxidants in serum, liver, brain, mammary gland)	
	Heat loss/m^0.75^	[+]	n.s.	+	+	+	Litter size, milk output, litter mass at weaning	M7
						n.s.	Female adult survival	
	BMR cov(m)	[+]	n.s.	+	+	−	Milk output, pup growth rate oxidative stress (damage to lipid in liver, kidney, heart; damage to DNA in serum) in nonreproducing females	M8
						n.s.	Litter size oxidative stress in reproducing females (same as above) humoral immune response	
	MMR cov(m)	n.s.	+ (20w)	n.s.		–	Response to inflammatory challenge	M9
						n.s.	humoral immune response	
Pig	FI (30 to 85 kg)		+	[+]		+	Litter size and mass	M10
						n.s.	Cellular immunity traits	
	FI/mass gain + fat thickness (30 to 85 kg)		n.s.	+		+	Litter size and mass	
	FI cov(mass gain, fat thickness) (35–95 kg)	+	n.s.	[+]	+	−	Litter size, litter mass gain to weaning response to hygiene challenge in growing males and females	M11
						n.s	Response to respiratory inflammation challenge	
						+	Oxidative stress (oxidative enzyme activity, gene expression of antioxidant in muscle) in growing females	
	FI cov(mass gain, fat thickness) (40 to 115 kg)	+	n.s.	[+]	+	−	Litter size	M12
						n.s.	Litter mass, piglet mass response to respiratory and enteric inflammation challenge	
						+/n.s.	Oxidative stress (mitochondria reactive oxygen species production in muscle (+) and liver (n.s.)) in growing females	
Cattle	FI cov(mass gain, m^0.75^) (28 to 54 w)	+	n.s.	+	+	n.s.	maternal productivity (fertility, weaning rate, calf mass at weaning)	M13

Note

Association between the selection criteria and other traits is rated as positive (+), negative (−) or not statistically significant (n.s.). Associations in square brackets ‘[]’ indicate direct responses to selection. RMR = resting metabolic rate; age is expressed in weeks (w), months (mo) or years (yr).

^a^ FI = feed intake; m = body mass; BMR/MMR = basal/maximum metabolic rate; FI cov(mass gain, fat thickness) adjustments of FI through covariance analysis where covariates include mass gain and fat thickness.

^b^ M1: Varkoohi et al. (2010); M2: Bordas et al. (1992), Gabarrou et al. (1998), Morisson et al. (1997), Zerjal et al. (2021); M3: Johnson and McLaury (1973), McLaury and Johnson (1972); M4: Pym and Nicholls (1979); M5: Brien et al. (1984), Brien and Hill (1986); M5: Brien et al. (1984), Brien nad Hill (1986); M6: Al Jothery et al. (2016), Hastings et al. (1997), Selman et al. (2001); M7: Bhatnagar and Nielsen (2014), Nielsen et al. (1997); M8: Książek and Konarzewski (2016), Sadowska et al. (2013); M9: Downs et al. (2013); M10: Clapperton et al. (2006), Kerr and Cameron (1995); M11: Chatelet et al. (2018), Gilbert et al. (2017); M12: Boddicker et al. (2011), Cai et al. (2008), Grubbs et al. (2013), Young et al. (2016), and M13: Arthur et al. (2005), Richardson and Herd (2004).

Although selection was supposed to be mass‐independent, about one third of the cases had a positive effect on body mass and several experiments showed an increasing mass of some digestive organs (e.g. liver mass; exps. M2, M6‐8 and M10; Table 3). In addition, experiments that measured physical activity all found it positively associated with feed intake. Altogether, these results support the ‘increased‐intake hypothesis’ (Biro & Stamps, 2010; Koteja, 2000; Nilsson, 2002), even when variation in body mass is accounted for.

We found equivocal evidence for a relationship between RMR metrics and components or physiological correlates of reproduction or survival (Table 3). Six of the 11 cases investigating those aspects detected a positive association between RMR and reproductive output, in agreement with previous results (Table 2). For instance, mice with high mass‐specific feed intake had large litter or high offspring growth in four experiments (exps. M5‐8). In contrast, in pigs, two experiments of divergent selection on the same criteria of residual feed intake (exps. M11‐12) reported the same negative effect on litter size, which suggests that mothers with low energy requirements for maintenance produce larger litters at least in early adulthood. Eight cases investigated a hypothetical physiological correlate of health, but did not support any clear link between responses to immune challenge and maintenance. Oxidative stress was the only marker that was positively related to residual or absolute feed intake in the four cases where it was studied (exps. M6 and M8 in mice, and exps. M11‐12 in pigs). Overall, from the limited data available and their potential bias (e.g. most observations were performed early in adulthood), our survey indicates that selection for feed efficiency involving low maintenance in early life does not simply result into a degradation of a fitness‐related trait. This parallels the lack of a consistent relationship observed at the phenotypic level between RMR and performance measures in the wild (Burton et al., 2011; Pettersen et al., 2018). Finally, it is noteworthy that the set of physiological traits commonly used to date to assess health and survival in that context (see Table 3) does not include accurate markers of survival prospects (Kennedy et al., 2014; López‐Otín et al., 2013), especially when they are investigated separately. For instance, field evidence from Soay sheep has revealed that multiple markers of oxidative stress are required to assess reliably the relationship between oxidative damage and survival (Christensen et al., 2016).

## 4 SHORTCOMINGS OF A RESOURCE SUPPLY–DEMAND APPROACH

**FIGURE 3:**
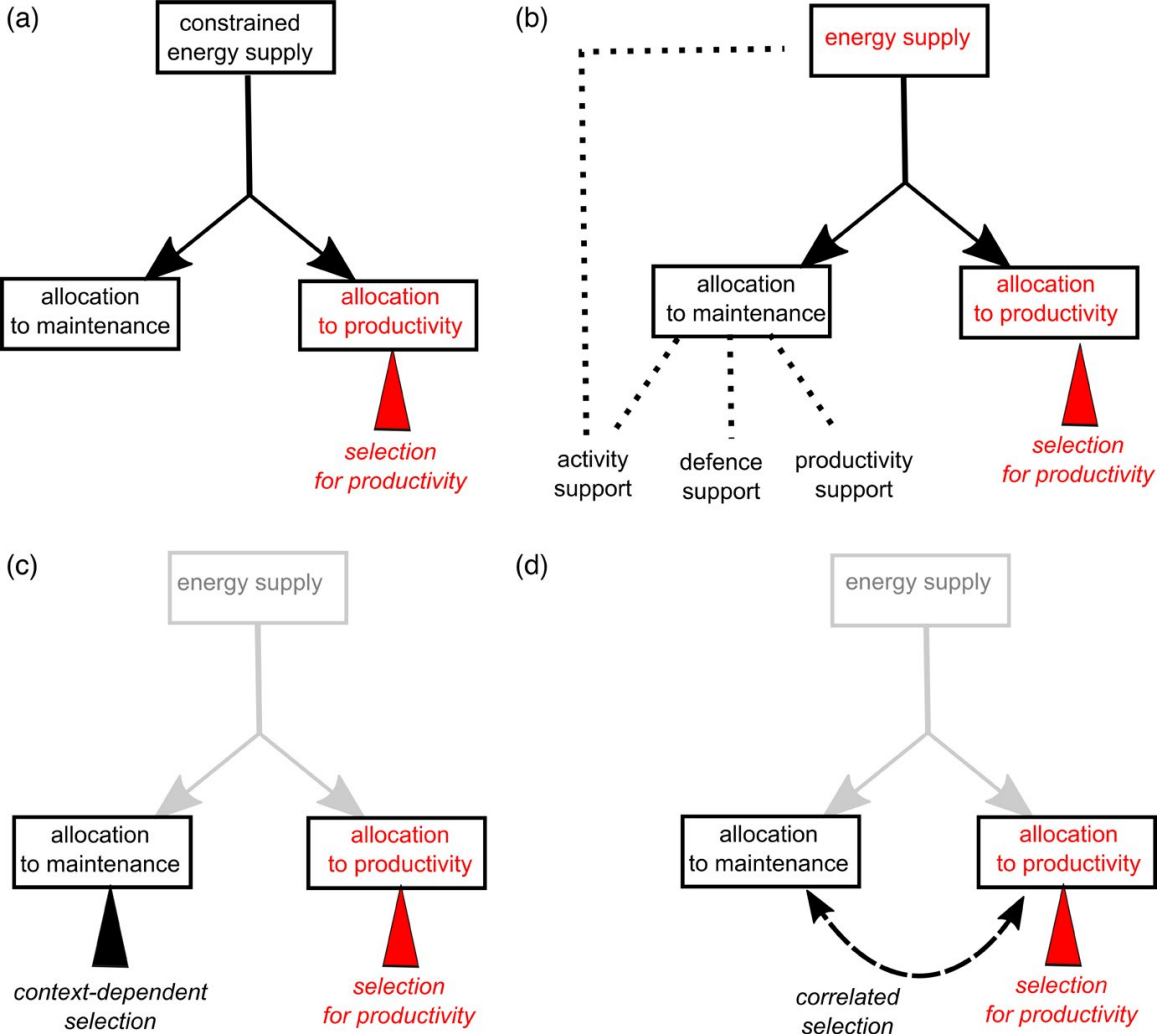
Pathways mediating the responses in energy allocation to maintenance to selection for increased productivity. (a) Allocation constraint under limited energy supply; (b) multifunctionality of maintenance, including foraging behaviour; (c) context‐dependent selection acting separately (e.g. selection for decreased antipredator response during domestication); and (d) correlated selection response in maintenance due to genetic association with the selected productivity trait. Pathways (a) and (b) involve physiological constraints on energy allocation (dark arrows), contrarily to (c) and (d) (grey arrows). Pathways are nonexhaustive and not mutually exclusive. Solid arrows indicate energy flows, dotted lines indicate functional relationships, dashed double arrows indicate genetic association, and triangles denote selective pressures

Trade‐offs in life history evolution are generally viewed as a result of competing allocation between functions for a fixed amount of resources. Although resource allocation trade‐offs have also been considered to play a key role in the context of livestock selection (Beilharz et al., 1993; Rauw, 2009), this application of the standard life history view (Figure 3a) lacks direct or mechanistic support as shown by our literature survey. In the following, we argue that this absence of identified pathway outlines several shortcomings of restricting the interpretation of the resource allocation framework in terms of a supply–demand approach. We discuss limitations to address the functional significance of among‐individual variation in the energy used for self‐maintenance (Section 4.1) and the premise that such variation stems from constraints on energy supply (Section 4.2). Alternatively, fitness costs of high energy intake may have been overlooked in livestock, as the focus on early‐life productivity provides little room for trade‐off detection later in life (Section 4.3). Accordingly, most previous assessments probably relied on inadequate data, arbitrary choice of biomarkers and heterogeneity in the timing of measurements (e.g. adult vs. old individuals). Those shortcomings concur with other limitations addressed from a life history perspective (Agrawal, 2020; Ng’oma et al., 2017; Speakman & Garratt, 2014).

### 4.1 Energy allocation to maintenance is difficult both to estimate and to interpret

In comparison with the energy expended in biomass production (e.g. milk, egg, meat, wool), the energy used for self‐maintenance and dissipated as heat is more challenging to estimate and to interpret functionally, particularly when animals are in a productive phase (Figure 3b). Just as RMR (Pettersen et al., 2018), feed efficiency is likely to involve more than one trait. Thus, using those metrics to measure energy allocation to maintenance as presented in Figure 1 is undoubtedly an oversimplification. For instance, in meat‐type chicken, a positive relationship between growth rate and RMR may translate into an increase in heat dissipated due to biosynthesis (proportional to growth rate) and, at the same time, a decrease in the energy needed for other functions, such as the expression of energetically expensive behaviours (Tallentire et al., 2016). The magnitude of the energy costs of biosynthesis is sometimes so high that it can flip the sign of the relationship between productivity and RMR if not discounted from total heat production (Konarzewski, 1995). From a physiological perspective, a dissection of the biological basis of whole‐organism measures of RMR (Cantalapiedra‐Hijar et al., 2018; Herd & Arthur, 2009; Konarzewski & Książek, 2013) does not simply translate into the resource acquisition–allocation framework. For instance, besides changes in energy acquisition and allocation, the efficiency of use in metabolizable energy in mitochondria is also subject to selection for high feed efficiency as observed in chicken (Bottje & Carstens, 2009), pigs (Gilbert et al., 2017) and cattle (Cantalapiedra‐Hijar et al., 2018). If high mitochondrial efficiency incurs fitness costs, notably through higher generation of reactive oxygen species (ROS; Salin et al., 2015), this would imply that fitness costs do not necessarily result from a low energy allocation to somatic maintenance *per se*. Surprisingly, studies in livestock found that ROS was low in lines selected for high feed efficiency (Table 3) and that energetically repairing processes are actually enhanced in efficient animals with a relatively low RMR (Labussière et al., 2015; Piekarski‐Welsher et al., 2018).

Beyond previous limitations, allocation constraints (Figure 3a) are not always actively involved in the physiological mechanisms underpinning selection responses. Indeed, *ad libitum* access to nutrient‐dense feed is commonly considered as a main reason for the absence of trade‐off (Metcalfe & Monaghan, 2013), in particular the lack of consistent relationship between RMR and reproductive output (Burton et al., 2011). However, benign conditions do not preclude the existence of energetic trade‐offs, and even favour them in some contexts (Figure 3c). Thus, trade‐offs between growth and immunity in intensive poultry systems (Van Der Most et al., 2011) could be favoured by heavy reliance on antibiotics and low pathogen pressure. The consequent relaxed selection on immunity could enable standing and *de novo* genetic variation underlying immunosuppression (Gering et al., 2019), notably through changes in growth‐related genes (Leshchinsky & Klasing, 2001). Domestication and intense selection for productivity have resulted in the downregulation of several energetically expensive behaviours that are no longer needed in farming conditions (e.g. ability to escape predators, fighting for access to mating, extensive foraging behaviour; Diamond, 2002; Rauw et al., 2017). More sedentary lifestyle and easier access to feed that characterize modern livestock species compared with their wild counterparts could also favour morphological evolution, in particular smaller energetically expensive organs such as brain (e.g. in chickens; Jackson & Diamond, 1996) or heart (in pigs; Van Essen et al., 2018). Correlated changes in energy metabolism are also supported at the level of tissues. For instance, selection for high feed efficiency in pigs has resulted in a higher proportion of fast‐twitch glycolytic fibres (Gilbert et al., 2017), whose low protein turnover can contribute to reduce overall maintenance metabolism.

Overall, the complexity of the genetic architecture underlying those coordinated modifications makes the consequences of a particular selection more difficult to predict than one could anticipate from the resource allocation framework alone. For instance, experimentally selecting ancestors of modern chicken for reduced fear of humans has resulted in an increase in RMR (Agnvall et al., 2015). Even in the absence of direct selection for growth, several anabolic pathways were upregulated in low‐fear chickens, just as observed in the modern chicken (Jackson & Diamond, 1996). Reciprocally, our results suggest that a reduction in activity is probably the most obvious side effect of selection for high feed efficiency (Table 3), in line with Rauw et al. (2017). In livestock, many selection experiments have been performed on traits not directly related to feed efficiency (e.g. behavioural traits or resistance to specific diseases). Unfortunately, correlated responses on whole‐organism energy metabolism and allocation have too rarely been assessed. Although such responses are challenging to measure, they would be insightful to discriminate between the role of correlational selection and constrained resource supply in causing trade‐offs (Figure 3d).

### 4.2 Constraints on energy supply and expenditure are elusive

If there is a constraint on feed intake (as in Figure 3a), should selection for higher productivity effectively result in a reduced amount of energy allocated to other traits? Support for this has been provided by earlier experiments in mice, where selection for growth under restricted feed conditions has resulted in a decrease in RMR (Falconer, 1960; McPhee et al., 1987; Nielsen & Andersen, 1987). In contrast, livestock selected for high productivity are often fed with abundant nutrient‐dense feed. Moreover, physiological limits to feed intake have rarely been of concern, except in the growing chicken (Jackson & Diamond, 1996; Konarzewski et al., 2000; Ricklefs, 1985).

Physiological limits to sustained energy intake and expenditure have been extensively studied in rodents (reviewed in Speakman & Król, 2011). The focus of these studies has shifted from central limitations—where energy is assimilated—to limitations at the tissue level—where energy is used (Hammond et al., 1996)—or at the whole‐organism level—where it is dissipated as heat (Speakman & Król, 2010). However, the role of those limits acting within individual is rarely considered in the context of individual variation although this is a critical issue for the manifestation of trade‐offs (Careau & Wilson, 2017; van Noordwijk & de Jong, 1986). In particular, few studies have addressed whether potential limits are absolute or can be overcome by selection (Garland & Carter, 1994). To this end, the development of mechanistic approaches to link physiological processes to selection responses (e.g. Box 2) is a promising avenue for future research, with key applications for livestock breeding.

An absolute limit on daily feed intake is currently suspected to cause a plateau in commercial selection for fast‐growing chicken (Tallentire et al., 2018). However, the underlying mechanisms of this limit remain elusive. For long, the primary focus has been on limits in the developmental capacity of central organs involved in energy supply to keep up with the selective increase in tissue demand (e.g. Dror et al., 1977). A nutritional imbalance is associated with the development of various metabolic troubles (e.g. cardiovascular diseases and musculoskeletal disorders (Julian, 2005) or muscle abnormalities (Soglia et al., 2018) in poultry; but see also Prunier et al. (2010) in pigs or Ingvartsen et al. (2003) in dairy cows). However, the general picture that has emerged from decades of research on the limits to sustained energy intake suggests that multiple constraints are involved, to different extents throughout development, and both from the supply side through the digestive and circulatory systems and from the demand side through peripheral tissues (Ricklefs, 2003; Speakman & Król, 2011). Moreover, ontogenetic processes governing nutrient supply and demand are amenable to selection as demonstrated by selection on the shape of growth curves in chicken (Mignon‐Grasteau et al., 1999). Hence, the incidence of several metabolic diseases related to an imbalance between supply and demand has been reduced through selection (e.g. notably in chicken with tibial dyschondroplasia (Wong‐valle et al., 1992) or pulmonary hypertension (Pavlidis et al., 1997)). In these cases, selection for reduced disease incidence also led to a decrease in growth that may well reflect that facing the boundaries of the physiological space is ineluctable. However, in several cases, the limits that trade‐offs impose on the simultaneous improvement of antagonistically related traits have been overcome by selecting for an index that combines those traits (e.g. Index = *b*_1_ *X*_1_ + *b*_2_ *X*_2_, where *b*_1_ and *b*_2_ are the selection weights applied to traits *X*_1_ and *X*_2_, respectively) or by selecting for a composite trait (Neeteson‐van Nieuwenhoven et al., 2013). For instance, Nielsen et al. (2013) showed that in sows, selection for high litter size after the early nursing period simultaneously increased litter size at birth and neonatal survival, whereas a trade‐off exists between these two latter traits. However, another trait may then become first limiting (e.g. number of sow teats) and then a new target of selection. Knowing to what extent simultaneous selection for increased demand and lower supply can be sustained and how this translates morphologically would be useful to clarify the importance of physiological constraints at an among‐individual level.

So far, the hypothesis that morphological constraints on organs involved in energy supply would limit selection for productivity has received little support (Schmidt et al., 2009; Zuidhof et al., 2014). Indirectly, supply organs such as the heart, the liver or the intestine seem to respond to selection for increased nutrient demand. Moreover, clear evidence supports the view that selection for increased productivity in nutrient‐rich environments has mostly been achieved through higher feed intake. This has been the key response in selection experiments for increased growth rate (e.g. reviewed in mice and rats (Roberts, 1979), in rabbits (Feki et al., 1996), in Japanese quails (Marks, 1978), in chickens (Dunnington et al., 2013), in turkeys (Nestor et al., 2008), or in sheep (Thompson et al., 1985)), as well as for increased litter size at birth in mice (Rauw et al., 2000) or in rabbits (Quevedo et al., 2006). The same phenomenon has likely occurred in commercial populations too. For instance, data from digestibility trials indicate that a doubling of milk yield in US dairy cows between 1970 and 2014 was accompanied by a 70% increase in dry matter intake (Potts et al., 2017).

**FIGURE 4:**
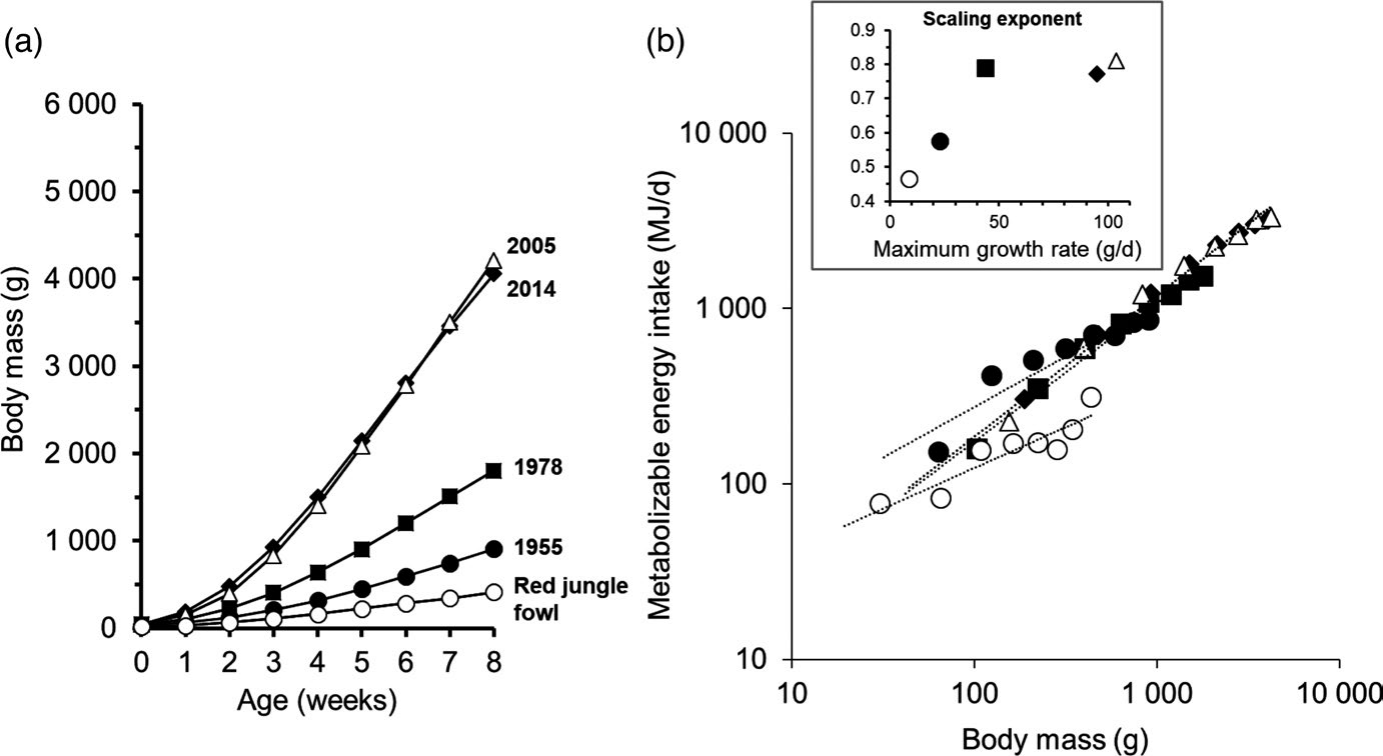
Selection for fast‐growing chicken has substantially changed growth curves and the allometric relationships between body mass and energy intake. Changes in chicken body mass during the first 8 weeks of age (a) are associated with changes in the scaling of energy intake with body mass (b). In inset, scaling exponents of the relations are reported according to the maximum growth rate of each curve shown in (a). When the metabolic costs of growth increase, the scaling exponent of energy intake converges towards unity. In other words, the growth of new tissue becomes proportional to existing tissue mass, as predicted by the metabolic‐level boundary hypothesis (Glazier, 2010). Data were compiled from Jackson and Diamond (1996) and Zuidhof et al. (2014) and from nutritional recommendations of a high‐yielding broiler chicken (Ross 308, Aviagen)

Overall, selection responses are also consistent with responses occurring within individuals as the achievement of increased growth rate through increased intake is also supported during ontogeny. For instance, in chicken, the ontogenetic scaling of MEI tends to be simply proportional to the increasing body mass (i.e. isometry) when chickens are increasingly fast‐growers (Figure 4). These changes in the scaling exponent thus suggest a continuous morphological adaptation when individuals with higher energy requirements are selected. This is in line with evidence showing that allometric relationships do not always resist selection (White et al., 2019). Nevertheless, the room for adaptation may be limited as proposed by the ‘metabolic‐level boundary hypothesis’ of Glazier (2010; Figure 4). Either way, those observations should warn against the application of interspecific rules of metabolic scaling within species and regardless of ontogeny in livestock nutrition (Noblet et al., 2015).

In our survey, support for the ‘independent’ or ‘increased‐intake hypothesis’ (paths 1 and 2, respectively, in Figure 1) suggests that despite intensive selection, resulting in a marked increase in nutritional requirements, the total energy budget is not constrained by an absolute limit on the rate of energy assimilation (as presented in Figure 3a). In the allocation framework, such physiological constraint would be a prerequisite for a negative association between productivity and maintenance (Konarzewski et al., 2000). Our results are thus in line with the rationale of most feeding recommendation systems that consider that more productive animals will simply eat more to meet their higher nutritional requirements (Emmans & Kyriazakis, 2001). However, livestock nutritionists generally consider that maintenance nutrient requirements are independent from the level of productivity (Johnson et al., 2003). Others have recommended to revise those requirements upwards to account for an additional demand accompanying increased intake. For instance, the high contribution of visceral organs to RMR is clear according to relationships between RMR and the mass of organs in cattle or in pigs (Ferrell, 1988; van Milgen et al., 1998). However, the mass of digestive organs is not always a good predictor of the energy costs of feed intake (Sadowska et al., 2013; Selman et al., 2001; Speakman et al., 2004).

### 4.3 Fitness costs of intake have been largely overlooked to date

Although physiological constraints on energy intake and expenditure remain elusive, this does not preclude a physiological restraint that is evolutionary shaped due to the fitness costs of a high intake (Speakman & Król, 2005). Such genetically determined restraint would thus represent a limit that can be overcome by artificial selection for high energy expenditure. Owing to the allometric relationship between feed intake and RMR, it is not surprising that fitness costs of high productivity have also been considered in the evolution of upper limits to metabolic rate (Drent & Daan, 1980; Peterson et al., 1990; Piersma, 2011). In particular, long‐term energy expenditures may be restrained by an ‘optimal working capacity’, that is a maximum level of metabolism that can be sustained and beyond which the mortality risk would become too high due to body reserve exhaustion, organ failure or wear‐and‐tear processes. As maximum levels of energy expenditure inevitably decline with the duration of the effort (Peterson et al., 1990) and since productivity traits are by definition constrained by time, then we could similarly assume optimal levels of livestock productivity for particular environments.

In livestock, evolutionary hypotheses on the control of feed intake have received some attention (Illius et al., 2002; Tolkamp & Ketelaars, 1992) but they had relatively little impact on the research done on energetic efficiency (Johnson et al., 2003). Two main reasons can be the limited scope for the expression of fitness costs of feed intake late in life in livestock (cf. Results Section 3.2), and the weak empirical and mechanistic evidence of a negative association between high productivity early in adulthood and late‐life reproduction or survival traits (e.g. Douhard et al., 2016; Theilgaard et al., 2007). Senescence (i.e. age‐specific decline in survival and/or reproductive performance; see Gaillard & Lemaître, 2020) seems ubiquitous in contexts as different as those of populations in the wild (Nussey et al., 2013) and highly productive livestock (Brody et al., 1926; Theilgaard et al., 2007). Yet, the trade‐off observed in wild populations between high reproduction in early adulthood and performance later in life (Lemaître et al., 2015) is challenging to assess in livestock given that most of those animals are rapidly slaughtered or replaced (Hoffman & Valencak, 2020). For example, dairy cattle, sheep, pigs or goats are commonly slaughtered before reaching 30% of their possible lifespan (Hoffman & Valencak, 2020). Accordingly, genetic correlations between productivity traits and longevity are often found inconsistent (Essl, 1998). However, longevity is not a proper metric of senescence, which rather corresponds to the declining performance with increasing age that can be analysed well before individuals have reached old ages (Gaillard & Lemaître, 2017; Kinzina et al., 2019). Moreover, the currently quite short lifespan of reproducing adults on farm is often economically suboptimal when the different costs of maintaining herd structure are correctly accounted for. For instance, in high‐producing dairy farms, cows should be kept on average 2 years longer than at present (i.e. five lactations instead of three) to compensate for the farm costs of breeding females before they reach sexual maturity and then develop their milk yield potential (which peaks at around four annual lactations; De Vries, 2020). Animals able to sustain their production over the long‐term are also increasingly relevant to address welfare and ethical concerns of breeding intensively and remove prematurely too many young animals (Oltenacu & Broom, 2010; Prunier et al., 2010). Improved opportunity to assess senescence patterns in livestock should thus take place in the near future. If early–late life trade‐offs also hold in livestock, then strong selection for early productivity (e.g. early age at first reproduction and high litter size) may have incidentally favoured rapidly ageing individuals (Mysterud et al., 2002, on domestic sheep, Grange et al., 2009 on horses), which improves the power of studying senescence prior to old ages. In the context of improving livestock welfare and the lifetime production of reproducing adults through lifespan extension, biomarkers involved in health and senescence such as the telomere dynamics or the age‐specific changes in the DNA methylation profiles are coming under scrutiny (Monaghan et al., 2018; Simpson & Chandra, 2021). As those biomarkers of biological age become increasingly accurate, they may have a strong interest in the early detection of animals with a high potential for long‐life performance. For instance, the relative leucocyte telomere length quickly declines after birth and is positively correlated to lifespan in high‐producing dairy cows (Seeker et al., 2018). As this trait is moderately heritable, it could thus be considered in future breeding objective to extend cow lifespan. Such biomarkers (see also Simpson and Chandra (2021) for a discussion on the use of the epigenetic clocks in that context) may benefit to a broader understanding of early–late life trade‐offs (Lemaître et al., 2015). So far, little is known about the respective fitness consequences of energy intake and its allocation to somatic maintenance in livestock.

Viewing the physiological control of feed intake as a target of selection is also in line with incentives to consider more explicitly signalling pathways in the resource allocation framework (Glazier, 2015; Ng’oma et al., 2017) and in behavioural evolution (Garland et al., 2016). Although the genetic basis of the control and orchestration of short‐term and long‐term regulations of energy metabolism has long been acknowledged by scientists working on livestock (Bauman & Currie, 1980), the associated endocrine pathways and their genetic basis are still largely unravelled. In some cases, the effects of artificial selection on the hormonal control of nutrient allocation during productive phases or of behavioural traits have been extensively studied, but their implications in terms of senescence are rarely considered. For instance, major changes in the somatotropic axis of dairy cows have been associated with selection for high milk yield (Lucy et al., 2009), whereas those hormones also have an evolutionary conserved role in modulating senescence (Bartke et al., 2013). In chicken, selection for growth has substantially altered neuroendocrine pathways, leading to hyperphagia and obesity in adults (Decuypere et al., 2010), which raises major issues in terms of management and animal welfare (D’Eath et al., 2009).

## 5 CONCLUSIONS AND FUTURE DIRECTIONS

Observations in livestock do not conflict with the compelling evidence that supports resource allocation in the broad sense—that is the fact that changes in energy and material apportioning are part of selection responses. As observed in our study, genetic changes in energy allocation to maintenance are clearer when feed intake is part of the selection objective than when selection is simply based on a productivity trait. However, livestock data question the nature and the importance of resource allocation constraints in the narrow sense (Glazier, 2009)—that is the assumption that resource limitation primarily causes trade‐offs that limit selection responses. In particular, we found little evidence that a reduction in the energy allocation to maintenance is at the basis of genetic antagonisms observed in livestock populations between productivity traits and traits related to health or future reproduction. As we discussed, such lack of evidence may well reflect limitations in the measurement of self‐maintenance or in the data available so far in livestock to assess costs of high productivity on fitness‐related traits. However, it becomes increasingly clear that the resource allocation framework narrowly defined in terms of energy and nutrient supply and expenditure is insufficient to explain trade‐offs so that a more complete picture requires the integration of time constraints (Lemaître et al., 2020) and alternative physiological causes (Harshman & Zera, 2007; Leroi, 2001; Ricklefs & Wilkelski, 2002; Speakman, 2008; Zera & Harshman, 2001). Besides, evolutionary biologists and physiologists are converging towards a shared understanding of the molecular pathways underlying trade‐offs (Flatt & Heyland, 2011; Mauro & Ghalambor, 2020; Ng’oma et al., 2017). We argue that such integrated framework could also benefit from a higher connection with research in comparative physiology and livestock breeding and genetics. This in turn could come with strong practical implications to orientate future breeding objectives.

In particular, recent evidence from experiments performed on laboratory organisms has revealed that somatic maintenance can be improved at no fitness cost when nutrient‐sensing pathway is downregulated during adulthood (e.g. Carlsson et al., 2021; Lind et al., 2019). These results, which challenge the ubiquity of the principle of allocation for explaining the decrease in fitness‐related traits in late life, suggest that selection for a certain level of gene expression during early life (e.g. a level of expression associated with a high productivity) might have detrimental effects in late life if the selected level of gene expression become suboptimal from mid‐adulthood onwards (Gems & de Magalhães, 2021; Maklakov & Chapman, 2019). However, this emerging theoretical framework (i.e. hyperfunction theory, developmental theory of ageing, early‐life inertia; see Carlsson et al., 2021; Maklakov & Chapman, 2019) opens the door to an experimental uncoupling of the trade‐off between productivity on the one hand and both health and survival in late life on the other hand but remains to be embraced in livestock studies.

**FIGURE 5:**
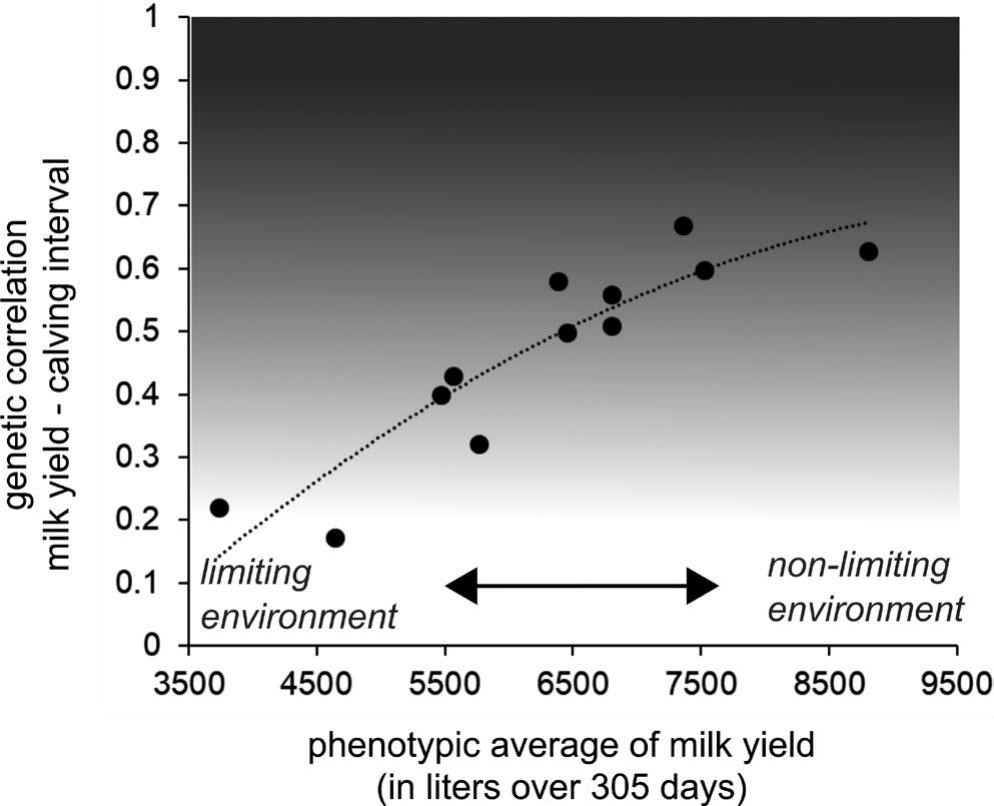
Genetic antagonism between dairy cow milk yield and fertility (calving interval, the time elapsed between two successive calving events) according to phenotypic average of milk yield (an indicator of energy availability in the production environment). Here, trade‐off intensity (grey shade) increases in less limiting environments. The polynomial trend is shown (dotted line). Figure adapted from Pryce et al. (2014)

Although livestock populations are often bred in relatively uniform and controlled environments, the large amount of data collected on these biological models offers a largely untapped opportunity to bridge research on physiological trade‐offs with population genetics. For instance, Schou et al. (2020) used estimates of micro‐environmental variance in dairy cattle and found some support for a stabilizing selection for milk yield in the most controlled environments. In other words, selection for productivity may have enforced an upper limit, possibly due to physiological constraints, and be then counterbalanced by natural selection. Another line of evidence for this hypothesis is suggested by published estimates of the genetic antagonism between dairy cow productivity (milk yield) and fertility (Berry et al., 2014). As shown by Pryce et al. (2014), this antagonism increases in the most favourable environments. Such relationship might be thus consistent with physiological constraints on high metabolic intensity (Figure 5). Would there be a link between such potential stabilizing selection in livestock populations and the concept of optimal working level of energy metabolism? Answers to this question will be favoured by framing the energy allocation to self‐maintenance as a multivariate trait in a microevolutionary framework so that genetic covariances among others traits can be measured and potential constraints identified (Konarzewski & Książek, 2013; Pettersen et al., 2018).

Finally, although continuous improvement in livestock productivity seems feasible from the perspective of quantitative genetics (Hill, 2016), there is now alarming evidence that industrial food systems that rely on this strategy are unsustainable given their contribution to unhealthy diets and environmental degradation (Willett et al., 2019). In other words, further increase in productivity may first be limited as a result of constraints at the level of livestock production systems rather at the level of animal biology. Some advocate further gains in animal productivity to reduce feed use and environmental impacts per unit of animal product (e.g. Capper & Bauman, 2013; Garnett et al., 2013). Yet, further increase in the genetic potential for productivity fuels the intensification and specialization of industrialized farming systems. In those systems, heavy reliance on external inputs (e.g. grains, freshwater, antibiotics) raise strong concerns in terms of global (e.g. greenhouse gas emissions, land conversion off‐farm) and local (e.g. soil and water pollution) footprints (Willett et al., 2019). Moreover, ethical debates exist on the acceptability of creating ever more productive livestock animals (Hötzel, 2014; Sandøe et al., 1999). Although industrial production systems have provided ideal conditions to foster continuous genetic gains in high‐yielding livestock (i.e. controlled and homogeneous environments), livestock breeding now needs to emphasize production in challenging environments (low levels of nutrition, heat stress; Hayes et al., 2013). In this context, the development of farming systems designed from ecological concepts and principles, as proposed by the agroecological paradigm (Altieri, 2018), provides a timely opportunity to renew the historical partnership between evolutionary biology and livestock breeding and genetics (Hill & Kirkpatrick, 2010). Agroecological systems seek to achieve whole‐system productivity by minimizing their reliance on external inputs, by using genetic diversity within farms and by promoting animal capacities to sustain lifetime production in varying conditions. In this respect, research on breeding strategies to improve animal capacities to recover from various environmental challenges (i.e. resilience) is at the forefront (Berghof et al., 2019), just like research on the flexibility of resource allocation and the plasticity of metabolic rate surges with climate change (Ng’oma et al., 2017; Norin & Metcalfe, 2019). Developing connections between those areas has great promise to address their integration within the resource allocation framework.

## CONFLICTS OF INTEREST

The authors declare no conflict of interest.

## Supporting information

Supinfo S1

## Data Availability

Data sharing is not applicable to this article as no new data were created or analysed in this study.
