# Supplementary material for: How much energetic trade‐offs limit selection? Insights from livestock and related laboratory model species
Source: Evol Appl. 2021 Nov 28;14(12):2726–49. doi: 10.1111/eva.13320 (PMC8674892; doi:10.1111/eva.13320)
Supplement: Supplementary file 1 — Supinfo S1 [file EVA-14-2726-s001.docx]

**Supplementary Material**

**Records identified from database searching**

Web of Science search (update August 2021)

(ALL=(metabolic rate OR maintenance OR heat production )) AND (ALL=(line OR breed OR strain)) and Multidisciplinary Sciences or Agriculture Dairy Animal Science or Genetics Heredity or Veterinary Sciences or Ecology (*Web of Science Categories*)

(n = 12126)

**Additional studies through other sources** (citation tracking, citation chaining using Google Scolar)

- **15** experiments comparing RMR between genotypes of contrasting productivity
- **4** selection experiments on feed efficiency

**Identification**

**Records remaining after non-relevant studies removed based on titles or abstract**

(n = 202)

**Screening**

**Records excluded:**

- no statistical comparison between genotypes (n= 7)

- genotypes not different for production or feed efficiency criteria (n = 54)

- RMR not measured or related metric not suitable (e.g. activity, feed restriction) (n = 77)

- study on embryo only (n = 2)

- study not accessible (n = 3)

(n = 143)

**Full-texts assessed for eligibility**

(n = 202)

**Eligibility**

**Eligible studies** (n = 59)

- **10** experiments comparing RMR between genotypes of contrasting productivity
- **9** selection experiments on feed efficiency

**Studies included in review**

- **25** experiments comparing RMR between genotypes of contrasting productivity
- **13** selection experiments on feed efficiency

**Included**

**Figure S1:** Search and selection processes of the reported experiments based on Preferred Reporting Items for Systematic Reviews and Meta-Analyses (PRISMA; Moher et al., 2009). RMR = resting metabolic rate

Moher, D., Liberati, A., Tetzlaff, J., Altman, D. G., & The, P. G. (2009). Preferred Reporting Items for Systematic Reviews and Meta-Analyses: The PRISMA Statement. PLoS Med, 6(7), e1000097. doi:10.1371/journal.pmed.1000097
